# Analyzing the Effects of Age, Time of Day, and Experiment on the Basal Locomotor Activity and Light-Off Visual Motor Response Assays in Zebrafish Larvae

**DOI:** 10.3390/toxics12050349

**Published:** 2024-05-09

**Authors:** Niki Tagkalidou, Cristiana Roberta Multisanti, Maria Jose Bleda, Juliette Bedrossiantz, Eva Prats, Caterina Faggio, Carlos Barata, Demetrio Raldúa

**Affiliations:** 1 Institute for Environmental Assessment and Water Research (IDAEA-CSIC), 08034 Barcelona, Spain; niki.tagkalidou@idaea.csic.es (N.T.); juliette.bedrossiantz@idaea.csic.es (J.B.); cbmqam@cid.csic.es (C.B.); 2 Department of Veterinary Sciences, University of Messina, Viale Giovanni Palatucci snc, 98168 Messina, Italy; cristiana.multisanti@studenti.unime.it; 3 Department of Chemical, Biological, Pharmaceutical and Environmental Sciences, University of Messina, Viale Ferdinando Stagno d’Alcontres, 31, 98166 Messina, Italy; cfaggio@unime.it; 4 Institute for Advanced Chemistry of Catalonia (IQAC-CSIC), 08034 Barcelona, Spain; mjbqbi@cid.csic.es; 5 Research and Development Center (CID-CSIC), 08034 Barcelona, Spain; eva.prats@cid.csic.es; 6 Department of Eco-Sustainable Marine Biotechnology, Stazione Zoologica Anton Dohrn, 80122 Naples, Italy

**Keywords:** zebrafish larvae, behavioral analysis, intrinsic factors, age, time of day, experiment, basal locomotor activity, light-off visual motor response

## Abstract

The recent availability of commercial platforms for behavioral analyses in zebrafish larvae based on video-tracking technologies has exponentially increased the number of studies analyzing different behaviors in this model organism to assess neurotoxicity. Among the most commonly used assays in zebrafish larvae are basal locomotor activity (BLA) and visual motor responses (VMRs). However, the effect of different intrinsic and extrinsic factors that can significantly alter the outcome of these assays is still not well understood. In this work, we have analyzed the influence of age (5–8 days post-fertilization), time of day (8:00, 10:00, 12:00, 14:00; 16:00, 18:00, and 20:00 h), and experiment (three experiments performed at different days) on BLA and VMR results (4004 analyses for each behavior) in 143 larvae. The results from both behaviors were adjusted to a random-effects linear regression model using generalized least squares (GLSs), including in the model the effect of the three variables, the second-way interactions between them, and the three-way interaction. The results presented in this manuscript show a specific effect of all three intrinsic factors and their interactions on both behaviors, supporting the view that the most stable time period for performing these behavioral assays is from 10:00 am to 04:00 pm, with some differences depending on the age of the larva and the behavioral test.

## 1. Introduction

Zebrafish (*Danio rerio*) is a model organism that has been widely used in different research areas, including developmental biology, pharmacology, neurobiology, and environmental toxicology [1,2,3]. This organism exhibits a remarkable resemblance at the genetic, molecular, and physiological levels with mammals, thus allowing the use of zebrafish as a vertebrate model in biomedicine studies [4,5]. In the neurotoxicology field, the high degree of similarity observed between zebrafish and mammalian nervous systems has led to the increasing use of zebrafish early larvae to predict the neurotoxic effects of environmental pollutants and drugs on vertebrates [6,7,8]. Since zebrafish larvae between 5 and 8 days post-fertilization (dpf) have an inflated swim bladder, well-developed sensorimotor systems, and a size of about 3.0–3.5 m [4], they are widely used for high-throughput analyses of neurobehavioral effects induced by chemical exposure [9]. Basal locomotor activity (BLA) and visual motor response (VMR), also known as the light/dark transition test (LDT), are the two more commonly used behavioral assays on zebrafish larvae using commercially available video-tracking methodologies, such as DanioVision (Noldus, Wageningen, the Netherlands) or ZebraBox (Viewpoint, Lyon, France) [8]. Locomotor activity in zebrafish early larvae is an essential behavior for starting exogenous feeding and is the result of coordinated brain, motor, and sensory pathways [10]. VMRs are used to assess the locomotive and visual skills of zebrafish larvae by exposing them to alternating periods of light and darkness (light-on and light-off VMRs) [11].

Research employing zebrafish behavioral methods encounters a significant obstacle, namely the inconsistency of findings within individual labs and across multiple research facilities. The absence of universally accepted testing procedures for assessing zebrafish behavior activities likely contributes to these variations in results among different labs [9]. To ensure the reliability and replicability of the results, it is important to recognize and define the intrinsic and extrinsic factors that influence larval behavior and its assessment [12]. Among the intrinsic factors, the age of the larvae and the time of day are known to strongly influence the outcome of the assays. The state of maturity of the neurotransmitter systems, the neuromuscular system, and the retinal OFF channels, the sensory circuit responsible for gathering visual stimuli, will increase with the age of the larvae. Therefore, age could affect the results of these behavioral assays [13,14,15]. In addition, the diurnal locomotor activity of larvae appears to be modulated by circadian rhythms, so the outcomes of the assays may be profoundly affected by the timing of the test [3,16]. Finally, different experiments could lead to some differences in the outcome of each assay due to the different batches of the larvae used.

In this study, our primary aim was to investigate how the age, time of day, experiment, and their interactions modulate larval behavior. To achieve this goal, 143 larvae were recorded from day 5 to day 8 post-fertilization in three independent experiments. During these four days, BLA and light-off VMRs were determined seven times a day (08:00, 10:00, 12:00, 14:00, 16:00, 18:00, and 20:00 h). The results presented here provide new information on the effects of these three intrinsic factors, and their interactions, on the results of BLA and VMRs. In addition, the most convenient periods for conducting each test, based on stability criteria, are proposed for each age.

## 2. Materials and Methods

### 2.1. Fish Husbandry and Larvae Production

Wild-type short-fin adult zebrafish were purchased from Pisciber (Barcelona, Spain) and maintained at the Research and Development Centre of the Spanish Research Council (CID-CSIC) facilities. Zebrafish embryos were obtained through natural mating, maintaining a female-to-male ratio of 3:2 in each mating tank. After collection, fertilized embryos were transferred into 6-well plates, with 20 embryos per well in 10 mL of fish water (MilliQ water containing 90 mg/L of Instant Ocean (Aquarium Systems, Sarrebourg, France); 0.58 mM CaSO_4_·2H_2_O; pH 6.5–7.0; and 750–900 μS/cm of conductivity), and kept at 28 °C ± 1 °C within a temperature-controlled chamber (POL-EKO APARATURA Climatic chamber KK350, Wodzislaw Slaski, Poland), following a 12L/12D cycle (light period: 08:00–20:00 h). Fish water was changed after the first 48 h. During the 5–8 dpf, zebrafish larvae undergo an endoexotrophic nutritional period in which they begin external feeding while continuing to consume residual yolk [17]. Since external feeding may be an additional source of variability, larvae were not fed throughout the experiment. All procedures received approval from the Institutional Animal Care and Use Committee (IACUC) at the CID-CSIC and complied with the institution’s guidelines, authorized by the local government under agreement N^o^ 11336.

### 2.2. Experimental Procedure

Following the criteria commonly used in developmental biology [18], the term “larva” was used across this manuscript for any zebrafish reared at 28 °C and older than 3 dpf (hatching period). According to the Directive 2010/63/EU on animal experimentation, however, a zebrafish reared at 28 °C is considered an “embryo” until 5 dpf, when this organism starts independent feeding. Therefore, the 5 dpf larvae tested in this study were considered eleutheroembryos from a regulatory point of view.

The experimental procedure used in this study is summarized in Supplementary Figure S1. Basically, 4 dpf zebrafish larvae were randomly transferred into a 48-well plate for the behavioral assessment. Each well contained one embryo in 1 mL of fish water. The plate was then covered with aluminum foil to prevent light disturbances and moved back into the temperature-controlled unit, ready to be used the next day. While handling the plate on the days of the experiment, the room was kept in darkness. After the last cycle, the plate was once again covered with aluminum foil and placed back inside the incubator. Throughout the experiment, from 5 to 8 dpf, the larvae remained in the same well. Fish water was introduced into the wells as needed to counteract potential evaporation and uphold water quality. This study took place from 5 dpf to 8 dpf, starting at 8 a.m. and ending at 8 p.m. each day. The behavior of each larva was assessed every 2 h. Behavioral testing was conducted in three different experiments, considering an experiment involving a microplate with 48 zebrafish larvae collected on the same day from the same spawn.

### 2.3. Behavioral Analysis

#### Behavioral Assessments in Zebrafish Larvae

Behavioral assessments in fish were carried out using the DanioVision platform (Noldus, Wageningen, the Netherlands), comprising a DanioVision Observation Chamber (DVOC) equipped with near-infrared illumination and a temperature control unit to maintain the system at 28 °C during all trials. Video tracking was conducted using EthoVision XT13 and behavioral analysis was carried out using Noldus EthoVision XT 16 software (Wageningen, the Netherlands). Recordings were made at a frame rate of 25 frames per second (fps). Larvae were allowed to acclimate in the DVOC for 60 min under dark conditions before the start of the recording. The routine started recording with a period of 15 min in dark conditions, and then, suddenly, a light phase (intensity: 100%; duration: 10 min) began. The light period was followed by a dark phase (intensity: 0%; duration: 95 min). Seven repetitions of this routine were performed. Then, a new trial of the assessment began. This resulted in obtaining data every 2 h, from 8 a.m. to 8 p.m. The EthoVision software tool’s multi-tracking module was employed to analyze the light-off visual motor response (VMR) and basal locomotor activity (BLA) for each individual larva. The VMRs and BLA were evaluated according to methods previously described by Bedrossiantz et al. [19]. Briefly, light-off VMRs were calculated by subtracting the distance covered during the first 2 min of darkness from the distance covered during the final 2 min of the preceding light period in centimeters. BLA was defined by calculating the total distance (cm) that the larvae travelled during a 10-min period in darkness. Following the tracking process, a quality control step was implemented, where wells displaying incorrect tracking were excluded from the final dataset.

### 2.4. Data Analysis

A total of 3 independent experiments (experiment 1:48 larvae, experiment 2:48 larvae, and experiment 3:47 larvae; each experiment was conducted on a different day) were analyzed in this study, giving a total number of 143 larvae. The BLA and light-off VMR of each larva was measured during 4 consecutive days, from 5 to 8 dpf. At each age, these behaviors were determined in each larva every 2 h during the whole daytime period (from 8 a.m. to 8 p.m.), giving a total of 7 measurements per larva and age. Therefore, each behavior was determined 4032 times in total. For some unknown reason, during the third replicate, Ethovision did not record the trials that were performed at 20:00; therefore, the total number of larvae tested at that time was 96 instead of 143.

### 2.5. Statistical Analysis

The data structure was longitudinal, with a series of 28 measurements per larva. Graphical and numerical descriptive results were calculated. The standard deviations (SDs) between larvae (over larvae) and within larvae (over days/hours) were calculated together with the Intraclass Correlation Coefficient (Rho). Normality tests and graphs were used to check the assumption of normal distribution of BLA and light-off VMR variables before adjusting a Random-Effects (RE) linear regression model [20], using generalized least squares (GLS), to take into account the clustering (correlation) of the measures of each larvae over time (over days/hours). This model included the main effect of variables day (age), time (hours), experiment (replicate), the second-way interactions between them, and the three-way interaction, as shown in Equation (1):

(1) $$y_{it}=\alpha +x_{it}\beta +\upsilon_{i}+\epsilon_{it}$$

where i=1,…,n, and for each *i*, t=1,…,T, of which $T_{i}$ periods were actually observed

$$x_{it}\beta =x_{1_{it}}\beta_{1}+x_{2_{it}}\beta_{2}+x_{3_{it}}\beta_{3}+\;+x_{1_{it}}x_{2_{it}}\beta_{4}+x_{1_{it}}x_{3_{it}}\beta_{5}+x_{2_{it}}x_{3_{it}}\beta_{6}+\;+x_{1_{it}}x_{2_{it}}x_{3_{it}}\beta_{7}$$

with $x_{1}$ = age (dpf), $x_{2}$ = time (hours), and $x_{3}$ = replicate being our categorical factors.

Intraclass Correlation of the error of the model (*ρ*) and within, between and global R-squared (squared correlation between original and fitted values of dependent variable) were calculated [20]. Once the goodness-of-fit of our statistical model was assessed, predictions (fitted values) were calculated, following which the fitted response obtained was able to be margined. A margin is a statistic based on the fitted model calculated over the dataset in which some or all of the covariates have been fixed. What are called margins of responses are also known as predictive margins, predictive marginal effects, adjusted predictions, or recycled predictions (when applied to balanced data, they are also called least squares means). Making the calculations of a predictive marginal effect, for each covariate the direct effect but also the indirect effects (interactions) were taken into account. Standard errors of predictive marginal effects were obtained by the delta method, and confidence intervals and test comparisons were determined. Pairwise comparisons were adjusted using the Bonferroni method. *p*-values ≤ 0.05 were considered statistically significant, and *p*-values between the interval (0.05;0.10) were considered borderline for statistical significance [21]. STATA 17.0 statistical softwarewas used to perform all statistical analyses [22].

## 3. Results

### 3.1. Basal Locomotor Activity (BLA)

**Figure 1:**
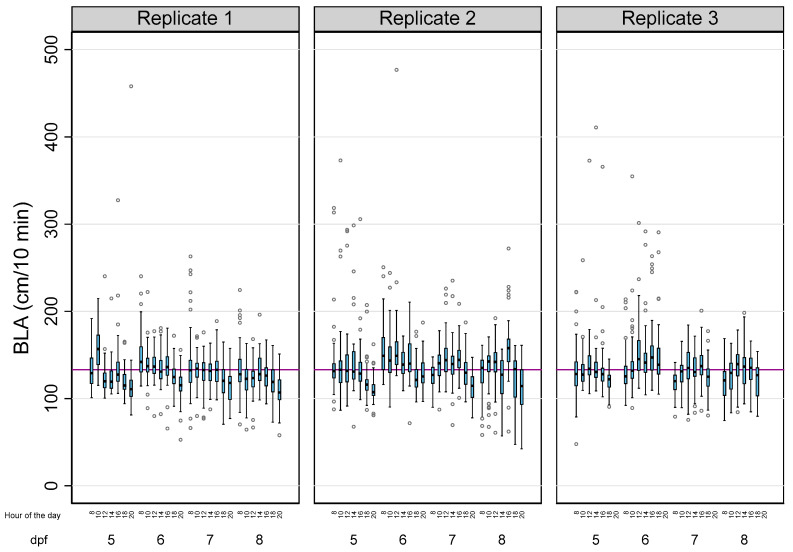
The effects of age, the time of the day, and the experiment on the basal locomotor activity (BLA) levels in zebrafish larvae. Boxplots indicate the 25th and 75th percentiles; whiskers show ±1.5 × IQR; and the center line within the box depicts the median. The purple line represents the BLA average (133.14 cm/10 min).

Table 1 summarizes the main descriptive results of 3814 analyses of BLA performed on a total of 143 zebrafish larvae in this study. Due to the tracking errors identified during the analysis of a few larvae, there was a small percentage of missing data (5.41%). The overall BLA of the larvae during the 28 reads was 133.14 ± 28.99 cm (mean ± SD), with 83.57% of the observed variability explained by intra-individual differences. When the effects of the age of the larvae, the time of the day, and the experiment were considered (Table 1 and Figure 1), the differences within each larva also explained most of the observed variability, with their percentages (1-Rho) ranging from 57.81% (5 dpf larvae) to 85.36% (larvae from the third replicate).

The BLA results were adjusted to the age of the larvae (5–8 dpf), the time of the day (08:00–20:00 h), the experiment (1–3), and the full interactions using a RE linear regression model; this model was able to explain 16.8% of the total variation (Supplementary Table S1).

Intra-individual differences (within) accounted for 18.84% of the variation, while the inter-individual differences (between) accounted for only 6.15%. The variable experiment had a big effect on the model, with the predictive margins of experiment 1 being statistically lower than experiments 2 (*p* = 0.004) and 3 (*p* = 0.018). Adjusting for this variable, the predictive margins of age were statistically different between all pairwise comparisons, except for the comparison between 5 and 7 dpf (*p =* 1.000), with the biggest difference (−13.25 cm/10 min) found between 6 and 8 dpf (95%CI: from −16.22 to −10.28) (Table S2). Finally, the predictive margins of the time of the day on the BLA levels of the larvae at 08:00 h were significantly lower than those at 12:00 h (*p* = 0.001) and 16:00 h (*p* = 0.000) but were significantly higher than those at 18:00 (*p* = 0.000) and 20:00 h (*p* = 0.000) (Table S3).

Next, the predictive margins of the time of the day on the BLA levels of the larvae, adjusted by the replicate effect, were determined. No differences were found in the BLA predictive margins between 10:00 and 16:00 h (*p* = 0.672). While the predictive margins of BLA between 16:00 and 18:00 h showed a significant (*p* = 0.000) decrease of −15.76 cm/10 min (95% CI: from −20.19 to −11.34), the greatest decrease, −24.60 cm/10 min (95%CI: from −29.86 to −19.34), was found between 16:00 and 20:00 h (*p =* 0.000).

**Figure 2:**
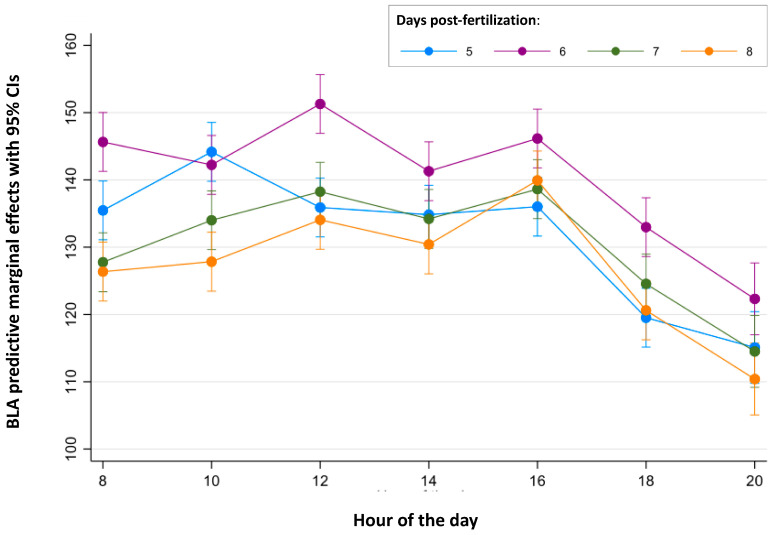
Predictive margins for basal locomotor activity (BLA) by the hour of the day in zebrafish larvae over 5 to 8 days post-fertilization. The error bars denote 95% confidence intervals.

**Figure 3:**
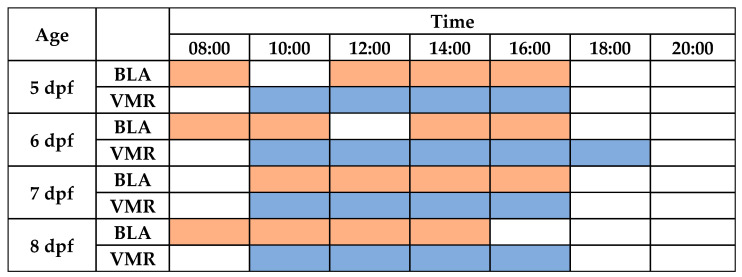
Diurnal periods with the highest stability for performing basal locomotor activity (BLA) and visual motor responses (VMRs) in zebrafish larvae. The shaded cells (orange for BLA or blue for VMR) denote that all predictive margins pairwise comparisons between times were not statistically significant.

Finally, the continuous periods of time in which BLA is stable were analyzed for each age (Figure 2 and Figure 3 and Table S4). For 5 dpf larvae, no differences were found between the BLA predictive margins at 8:00 and during the period from 12:00 to 16:00 h. For 6 dpf larvae, BLA predictive margins were similar from 08:00 to 10:00 h and from 14:00 to 16:00 h. BLA predictive margins at 12:00 h were only 7% higher than those at 14:00 h (141.29 cm and the minimum value of this period), but this difference was found to be statistically significant (*p* = 0.012). For 7 dpf larvae, BLA predictive margins were similar during the period from 10:00 to 16:00 h. Finally, for the 8 dpf larvae, their BLA predictive margins were similar between 08:00 and 14:00 h.

### 3.2. Light-Off Visual Motor Response (VMR)

**Figure 4:**
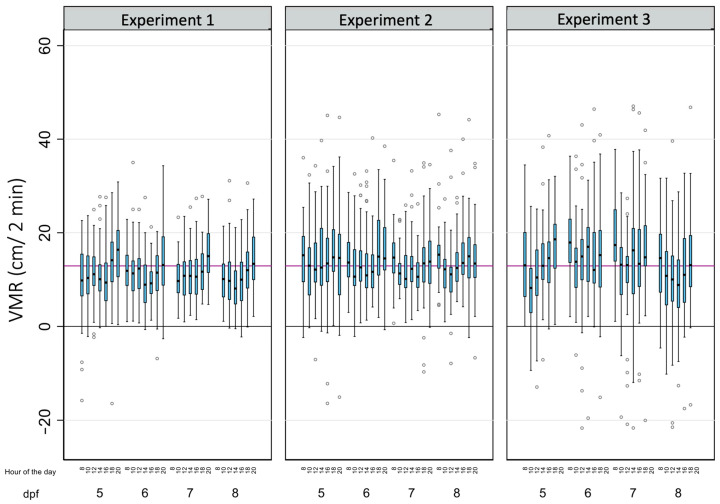
The effects of age, the time of the day, and the experiment on the light-off visual motor response (VMR) in zebrafish larvae. Boxplots indicate the 25th and 75th percentiles; whiskers show ±1.5 × IQR; and the center line within the box depicts the median. The purple line represents the BLA average (12.91 cm), and the black line denotes the zero value.

Table 2 summarizes the main descriptive results of 3699 analyses of light-off VMRs performed on a total of 143 zebrafish larvae in this study. As for BLA, due to the tracking errors identified in a few wells during some reads, there is a small percentage of missing data (8.26%). The average light-off VMR was 12.79 ± 8.56 cm, with 81.86% of the observed variability explained by the differences within each larva. When the effects of the age of the larvae, the time of the day, and the experiment were considered (Table 2 and Figure 4), the differences within each larva also explained most of the observed variability, with their percentages ranging from 50.91% (8 dpf larvae) to 86.43% (larvae from the third replicate).

When the light-off VMR results were adjusted to the age, time of the day, experiment, and their full interactions using the RE linear regression model, the model was able to explain 8.4% of the total variation (Supplementary Figure S2 and Table S1). Intra-individual differences accounted for 8.17% of the variation, while inter-individual differences accounted for 8.95%. The variable experiment had a big effect on the model, with the predictive margins of replicate 1 being statistically lower than those of replicate 2 (*p* = 0.000) and 3 (*p* = 0.001). Adjusting for this variable, the predictive margins of VMRs in 8 dpf were statistically lower when pairwise comparisons with 6 dpf (*p* = 0.000) and 7 dpf (*p* = 0.024), with the biggest difference (−1.29 cm) found between 6 and 8 dpf (95%CI: −2.14 to −0.44) (Table S5). Finally, the predictive margins of the time of the day on the light-off VMR levels of the larvae, adjusted by the replicate effect, were determined. While no differences in the predictive margins were found between 10:00, 12:00, 14:00, and 16:00 h, and between 8:00, 18:00, and 20:00 h (*p* =0.423), an increase of 3.52 cm was found between 10:00 and 20:00 h (95%CI: from 2.04 to 4.99) (Table S6).

**Figure 5:**
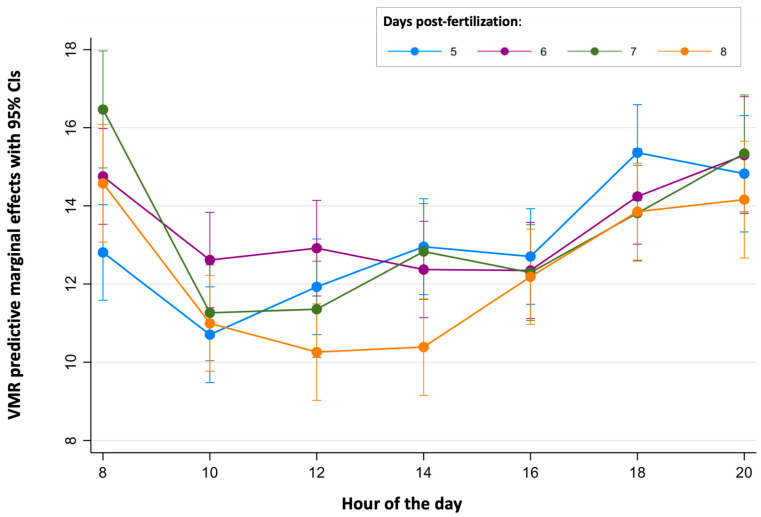
Predictive margins for light-off visual motor response (VMR) by hour of the day in zebrafish larvae over 5 to 8 days post-fertilization. The error bars denote 95% confidence intervals.

Finally, the continuous periods of time where the light-off VMRs were stable were determined for each age (Figure 3 and Figure 5 and Table S7). For 5 dpf larvae, no differences were found between the light-off VMR predictive margins during the period of time from 10:00 to 16:00 h. For 6 dpf larvae, light-off VMR predictive margins were similar from 10:00 to 18:00 h. For the 7 dpf and 8 dpf larvae, their light-off VMR predictive margins were similar in both ages between 10:00 and 16:00 h.

## 4. Discussion

In this work, we have shown that several intrinsic variables significantly affect the locomotor behavior of zebrafish larvae. When locomotor behavior is used to assess the neurotoxicity of one chemical or a chemical mixture, it is assumed that any changes in behavior are due to the chemical treatment. However, we have seen here that the locomotor behavior of 5–8 dpf zebrafish larvae is extremely sensitive to different factors other than chemical treatment, and if these variables are not conveniently controlled, they can lead to the generation of type 1 and 2 errors. While different studies have already reported a significant effect of the age of the larvae on BLA, this effect seems to be modulated by the interactions with other intrinsic and extrinsic factors. Celine de Esch et al. found that the effect of the age on BLA in larvae from 5 to 7 dpf was strongly dependent on the strain (5 dpf> 6 dpf > 7 dpf for AB strains; 7 dpf > 5 dpf ≈ 6 dpf for TL strains) [23]. In our study, however, the population of wild-type adult zebrafish (blue-fin-short phenotype) maintained at the CID-CSIC is genetically heterogeneous and therefore more representative of the variability of natural populations than wild-type laboratory strains [24,25]. Moreover, there are differences in the size of the experimental arena between de Esch’s study (96-well microplates) and ours (48-well microplates), and this extrinsic factor has been reported to modify the outcomes of both BLA and VMRs [12]. Therefore, differences in the zebrafish strain and experimental arena size could lead to the differences observed between de Esch’s study and the results presented in this manuscript (6 dpf > 5 dpf ≈ 7 dpf > 8 dpf). Ingebretson et al. found that 4 dpf larvae exhibited significantly higher BLA levels than 7 dpf larvae [26]. Again, the differences in the experimental conditions used by Ingebretson and ours (different well diameters for 6-well vs. 48-well microplates; light vs. dark conditions; 4 dpf vs. 5 dpf larvae; and one measure vs. seven measures per day) make very difficult, if possible, a direct comparison of the results. There are different factors that could explain the low BLA observed in the 7 and 8 dpf larvae compared to the 5–6 dpf larvae in our study. From 5 dpf, zebrafish larvae are actively searching for food (food-seeking behavior), so they exhibit high locomotor activity during the daytime. At 7–8 dpf, zebrafish larvae could exhibit a mitigation in the food-seeking behavior due to the fact that they have not found food in their environment [27,28]. In addition, the decrease in BLA could be related to a decrease in energy supply in the older larvae, as by 7–8 dpf, the yolk sac is almost depleted of nutrients [29]. Hurd and Cahill (2002) demonstrated that zebrafish embryos trained to 14:10 LD from the first 3 days of development and then transferred to DD conditions until 8 dpf were able to maintain their circadian rhythms during all these periods [30]. Therefore, we can discard that the decrease in BLA observed in 7–8 dpf larvae in this study is related to a disruption of the circadian rhythms resulting from the experimental design used.

Regarding the effect of the time of day on BLA performance, MacPhail et al. reported, in 6 dpf larvae, an initial decrease in BLA levels from 10:00 to 13:00 h, followed by a period of stability between 13:00 and 15:30 h [31]. In MacPhail’s study, 6 dpf larvae were raised at 26 °C, and considering the algorithm proposed by Kimmel to compare the developmental stage of embryos raised at different temperatures, 144 hpf (6 dpf) larvae reared at 26 °C are equivalent to 124 hpf (about 5 dpf) larvae reared to the standard 28.5 °C [18]. Therefore, when we compare the results by MacPhail with those presented in this manuscript for 5 dpf larvae, despite their differences in photoperiod (14:10 vs. 12:12) and the arena size (98 wells vs. 48 wells), there is a period of stability between 13:00 and 16:00 h. Finally, Kristofco et al. found that for 5–8 dpf larvae, their level of BLA at 09:00 was significantly lower than at other times of the day, increasing thereafter until 12:00 h, and reaching their highest stability during the period 14:00–18:00 h [32]. Again, the different experimental conditions used in both studies could be behind the observed differences in the modulatory effect of the time of the day on BLA, which emphasizes the urgent need for harmonizing the conditions of the assay (e.g., strain, embryo rearing conditions, experimental arena size, time of day, lighting conditions, etc.) in order to make it reproducible among laboratories.

The sudden decrease in light intensity triggers an acute locomotor response in zebrafish larvae [13], also known as the light-off VMR. The sensory circuit involved in this response is the OFF channel of the retina, which, in response to a sudden decrease in illumination, transmits signals from photoreceptors to specific OFF ganglion and bipolar cells and ultimately to higher visual centers [14]. Activating this circuit evokes the O-turns, followed by an hyperactivity period [33]. The light-off VMR is the most relevant component of the light/dark transition test (LDT), one of the most frequently used behavioral assays with zebrafish larvae [34]. This assay is generally based on measuring the locomotor response induced with a series of alternating light and dark periods [35]. There is a great variability in the number and duration of light and dark periods of the LDT between laboratories [23,31,36,37,38,39,40]. In addition to these extrinsic factors, some intrinsic factors, such as age and time of day, have also been shown to modulate the outcomes of the LDT [12,32,35]. While in our study, 6 dpf larvae exhibited higher light-off VMRs than 5 dpf larvae, Stephanie Padilla et al. found that the light-off VMR was higher for 5 dpf larvae than 6 dpf larvae [12]. The differences in the rearing (26 °C vs. 28 °C; 14L:10D vs. 12L:12D photoperiod) and the size of the experimental arena (96 wells vs. 48 wells) could explain the differences observed between both studies. In contrast to the differences observed with the age in the VMR in our study, no differences were found by Jennifer A. Fitzgerald et al. [38]. However, the differences in the rearing conditions (26 °C vs. 28 °C; 14L:10D vs. 12L:12D photoperiod) and the effect of the time of day (09:00 vs. 14:00 h) make it difficult to carry out a direct comparison. As already discussed for BLA, there is an urgent need to harmonize the LDT protocol in order to allow the comparison of results across laboratories.

A possible objection to the experimental design used in the present study is that BLA and MRV were analyzed in each larva 28 times in total, so in most of the trials, the larvae were not experimentally naïve. However, neither motor activity, as measured by the BLA, nor the escape reflex evoked by a visual stimulus, measured by the VMR, are dependent on novelty. Larvae were placed in a multi-well plates at 4 dpf, and from days 5 to 8, the plate was placed 12 h per day inside the DVOC. During these 12 h, the larvae were unaware as to when they were being recorded. Habituation is a progressive decrease in responses when a series of stimuli are presented to the same animal [41]. However, with our design, the possibility of habituation with the visual stimuli can be ruled out, as the interstimulus interval is of 2 h, and habituation only happens when the interstimulus interval is very short, of the order of 1 s [41].

There is a lack of studies analyzing the effect of the experiment on the outcomes of behavioral analysis in zebrafish larvae. The results presented in this study show, however, that the experiment is an important intrinsic factor of variability for BLA and VMRs, both alone or in combination with age or the time of day. Further efforts should be made to better understand the effect of this factor on behavior.

It is important to note that the experimental design used in this work has some potential pitfalls. Keeping each larva in the same well for 5 days (4–8 dpf) could potentially result in an accumulation of degradation products in the well. It is also difficult to predict whether repeating the light off-VMR seven times a day for 4 days may have an effect on the outcome of the assay. The alternative to avoid these pitfalls would be to use experimentally naïve animals for each behavioral measurement. However, using experimentally naïve animals in this study would have meant using 4023 animals (48 animals × 7 measures per day × 4 days × 3 replicates) instead of the 143 that were actually used, something completely against the 3Rs principle. It should be noted that the majority of this study has been conducted with larvae older than 120 hpf, so, from a regulatory point of view, these larvae are considered experimental animals. Therefore, the design used takes very much into account the 3Rs reduction principle, and has the additional advantage of being able to provide information on variability both for the same animal and between animals.

## 5. Conclusions

The first conclusion of this study is that researchers conducting behavioral analyses in neurotoxicity studies should be aware of all the intrinsic and extrinsic factors that potentially modulate zebrafish behavior. Second, the stability periods for the BLA and light-off VMRs identified in this study will be only useful for studies using experimental conditions similar to those described here. Third, more efforts should be invested in the study of the effect of the different experiments on the outcome of the assays. Finally, the data presented in this manuscript emphasize the urgent need to harmonize the conditions for conducting behavioral assays with zebrafish larvae.

## Figures and Tables

**Table 1:** Descriptive results of the basal locomotor activity (BLA) in zebrafish larvae from 5 to 8 days post-fertilization determined every 2 h during the 12 h of the diurnal period (08:00–20:00 h).

	*N*	*n*	$\bar{x}$	SD	SD_Between_	SD_Within_	Rho	1-Rho
	**3814**	**143**	**133.14**	**28.99**	**11.75**	**26.51**	**16.43%**	**83.57%**
***Day (age)***								
5	954	143	132.38	34.42	22.36	26.17	42.19%	57.81%
6	954	143	141.15	30.20	19.28	23.55	40.13%	59.87%
7	952	143	131.09	22.99	14.10	18.21	37.48%	62.52%
8	954	143	127.91	25.31	16.37	19.31	41.82%	58.18%
***Time (hours)***								
08:00	572	143	133.81	30.30	18.07	24.36	35.51%	64.49%
10:00	572	143	137.06	27.94	16.83	22.34	36.20%	63.80%
12:00	572	143	139.87	31.68	19.33	25.14	37.15%	62.85%
14:00	572	143	135.18	26.78	15.03	22.19	31.43%	68.57%
16:00	571	143	140.22	28.60	17.12	22.97	35.71%	64.29%
18:00	571	143	124.46	22.63	12.67	18.77	31.29%	68.71%
20:00	384	96	115.58	26.32	14.38	22.09	29.76%	70.24%
***Experiment***								
1	1344	48	129.17	25.77	9.89	23.84	14.67%	85.33%
2	1342	48	135.80	31.14	13.02	28.36	17.40%	82.60%
3	1128	47	134.69	29.45	11.28	27.25	14.64%	85.36%

Notes: *N* = number of measurements; *n* = number of larvae; $\bar{x}$ = mean; SD = standard deviation; Rho = % of the variability of measurements of BLA due to the differences between larvae; and 1-Rho = % of the variability of measurements of BLA due to the differences within each larva.

**Table 2:** Descriptive results of the light-off visual motor response (VMR) in zebrafish larvae from 5 to 8 days post-fertilization determined every 2 h during the 12 h of the diurnal period (08:00–20:00 h).

	*N*	*n*	$\bar{x}$	SD	SD_Between_	SD_Within_	Rho	1-Rho
	**3699**	**143**	**12.79**	**8.56**	**3.65**	**7.76**	**18.14%**	**81.86%**
***Day (age)***								
5	951	143	12.90	8.75	5.14	7.13	34.14%	65.86%
6	950	143	13.33	8.54	5.66	6.50	43.10%	56.90%
7	900	143	12.96	8.05	5.44	6.11	44.26%	55.74%
8	898	143	11.92	8.82	6.27	6.39	49.09%	50.91%
***Time (hours)***								
08:00	473	143	14.45	7.67	4.96	6.05	40.16%	59.84%
10:00	570	143	11.28	7.46	4.42	6.02	35.04%	64.96%
12:00	568	143	11.59	8.28	4.45	6.98	28.85%	71.15%
14:00	567	143	11.94	9.34	5.22	7.76	31.20%	68.80%
16:00	571	143	12.13	8.61	5.15	6.94	35.51%	64.49%
18:00	569	143	14.18	9.60	5.61	7.80	34.12%	65.88%
20:00	381	96	14.91	7.57	5.03	5.68	43.91%	56.09%
***Experiment***								
1	1244	48	11.41	6.21	2.83	5.55	20.61%	79.39%
2	1341	48	13.75	7.29	3.41	6.46	21.73%	78.27%
3	1114	47	13.17	11.53	4.26	10.74	13.57%	86.43%

Notes: *N* = number of measurements; *n* = number of larvae; $\bar{x}$ = mean; SD = standard deviation; Rho = % of the variability of measurements of BLA due to the differences between larvae; and 1-Rho = % of the variability of measurements of BLA due to the differences within each larva.

## Data Availability

Data supporting the findings of this study are available within the manuscript and its Supplementary Materials file, or will be available from the corresponding author upon request.

## Supplementary Materials

The following supporting information can be downloaded at: https://www.mdpi.com/article/10.3390/toxics12050349/s1, Supplementary Figures: Figure S1. Experimental procedure used for the validation of the basal locomotor activity and light off-visual motor response in zebrafish larvae; Figure S2. Understanding the 3-way interaction between age, hours, and replicate for BLA and VMR in the RE linear regression model. Supplementary Tables: Table S1. Random-effects (RE) linear regression model estimation using generalized least squares (GLS) for BLA and VMR variables; Table S2. Pairwise comparisons of predictive margins of BLA for age; Table S3. Pairwise comparisons of predictive margins of BLA for time of the day; Table S4. Pairwise comparisons of predictive margins of BLA for the time of the day and age; Table S5. Pairwise comparisons of predictive margins of VMRs for age; Table S6. Pairwise comparisons of predictive margins of VMRs for the time of the day; Table S7. Pairwise comparisons of predictive margins of light-off VMR for time of the day and age.
