# Supplementary material for: Analyzing the Effects of Age, Time of Day, and Experiment on the Basal Locomotor Activity and Light-Off Visual Motor Response Assays in Zebrafish Larvae
Source: Toxics. 2024 May 9;12(5):349. doi: 10.3390/toxics12050349 (PMC11125988; doi:10.3390/toxics12050349)
Supplement: Supplementary file 1 [file toxics-12-00349-s001.zip › toxics-2920476-supplementary.pdf]

Supplementary Material

Analyzing the effect of age, time of the day and the experiment on the basal locomotor activity and light-off visual motor response assays in zebrafish larvae

Niki Tagkalidou, Cristiana Roberta Multisanti, Maria Jose Bleda, Juliette Bedrossiantz, Eva Prats, Caterina Faggio, Carlos Barata, Demetrio Raldúa

Supplementary Figures

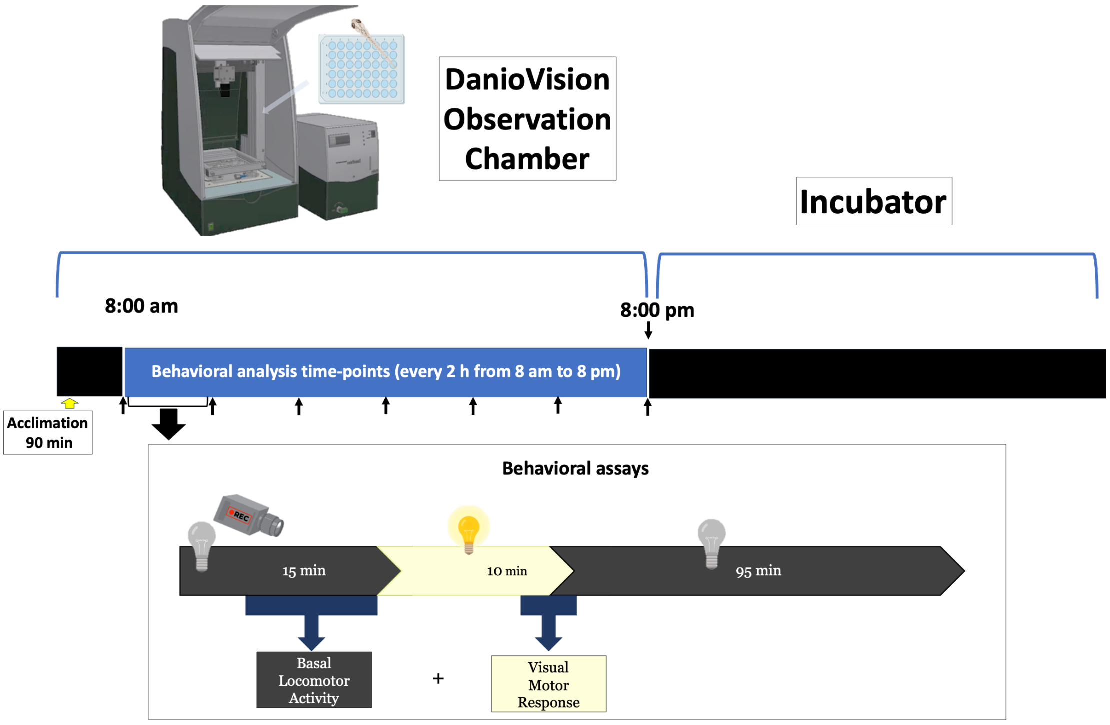

Figure S1. Experimental procedure used for the validation of the basal locomotor activity and light off-visual motor response in zebrafish larvae. The night before the validation experiment, zebrafish larvae were transferred to an incubator at 28°C in the dark (from 8 pm to 6:30 am under our conditions). On the day of the experiment, 90 min before the start of recording (8 am), the microplate containing the larvae was transferred to a DanioVision Observation Chamber for acclimation, where it remained at 28°C throughout the 12-hour procedure (from 8 am to 8 pm). A task consisting of 15 min dark/ 10 min light/ 95 min dark was programmed with Ethovision software. This task sequence was repeated 7 times. Upon completion the plate was transferred back to the incubator and the whole process was repeated on the subsequent days.

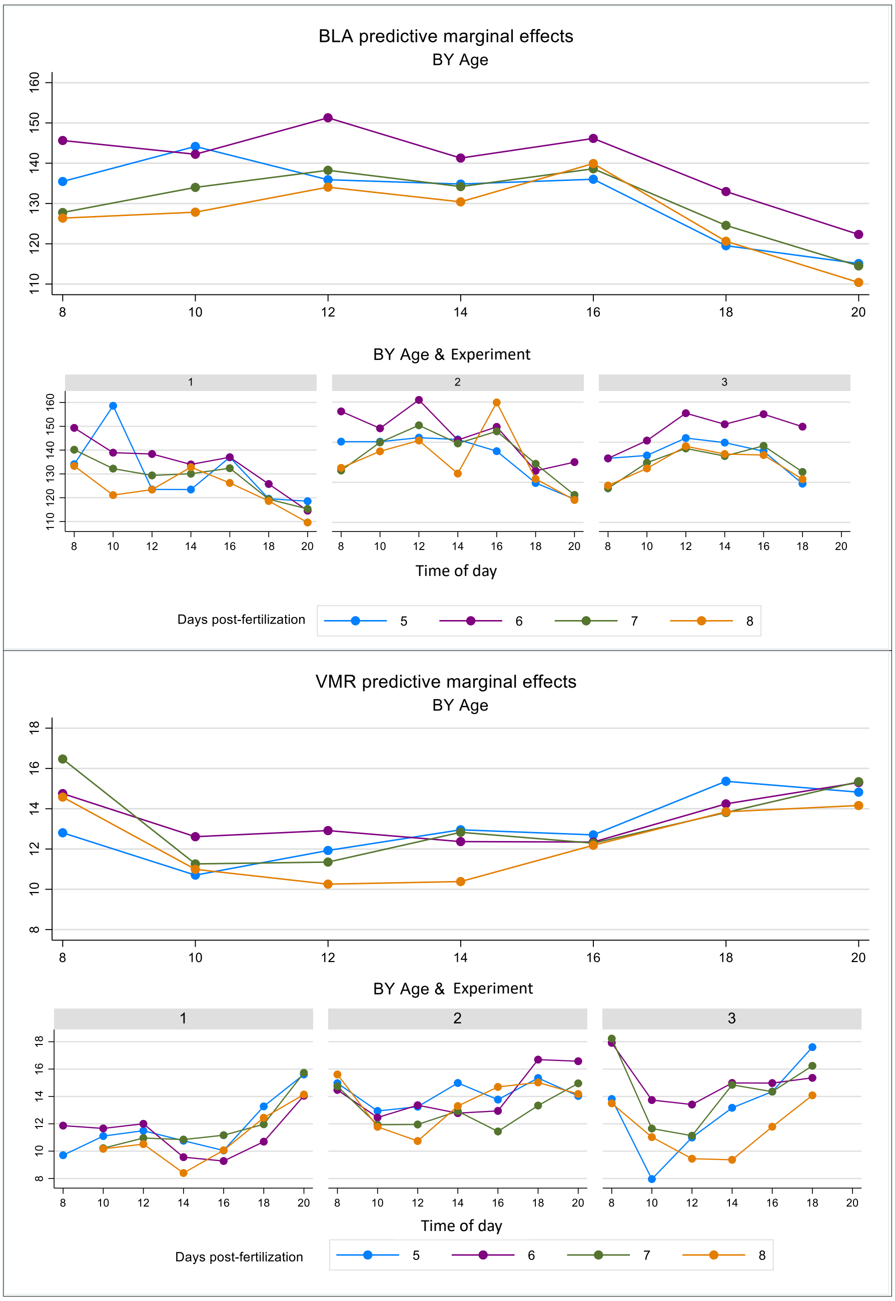

Figure S2. Understanding the 3-way interaction between age, hours and the experiment for BLA and light-off VMR in the RE linear regression model.

Supplementary Tables

Table S1. Random-Effects (RE) linear regression model estimation using generalized least squares (GLS) for BLA and light-off VMR variables. Main effects, full two-way interactions and the three-way interaction included.

Variables	BLA Coef.		BLA (95% CI)	VMR[†] Coef.		VMR[†] (95% CI)
**Age [days]**						
5	0.73		(-9.12 ; 10.57)	-5.78	*	(-11.33 ; -0.24)
6	15.98	*	(6.14 ; 25.83)	-3.62		(-8.42 ; 1.18)
7	6.80		(-3.04 ; 16.65)	-0.06		(-4.86 ; 4.74)
8 (Ref.)						
**Time [hours]**						
8:00 (Ref.)						
10:00	-12.24	*	(-22.09 ; -2.40)	-5.31	°	(-10.86 ; 0.23)
12:00	-9.93	*	(-19.78 ; -0.09)	-4.97	°	(-10.52 ; 0.58)
14:00	-0.52		(-10.36 ; 9.33)	-7.08	*	(-12.63 ; -1.54)
16:00	-7.14		(-16.99 ; 2.70)	-5.42	*	(-10.97 ; 0.13)
18:00	-14.71	*	(-24.56 ; -4.87)	-3.04		(-8.60 ; 2.51)
20:00	-23.75	*	(-33.59 ; -13.90)	-1.35		(-6.15 ; 3.46)
**Experiment**						
1 (Ref.)						
2	-6.14		(-16.81 ; 4.53)	0.12		(-4.82 ; 5.06)
3	-14.96	*	(-25.69 ; -4.23)	-1.99		(-7.66 ; 3.69)
**Day # Time**						
5 10:00	36.78	*	(22.85 ; 50.70)	6.71	*	(0.52 ; 12.91)
5 12:00	-0.66		(-14.58 ; 13.27)	6.76	*	(0.57 ; 12.96)
5 14:00	-10.12		(-24.04 ; 3.81)	8.14	*	(1.94 ; 14.34)
5 16:00	10.07		(-3.86 ; 23.99)	5.77	°	(-0.43 ; 11.97)
5 18:00	0.21		(-13.71 ; 14.14)	6.62	*	(0.41 ; 12.82)
5 20:00	8.21		(-5.71 ; 22.13)	7.25	*	(1.71 ; 12.79)
6 10:00	1.83		(-12.09 ; 15.75)	5.11	°	(-0.43 ; 10.65)
6 12:00	-1.04		(-14.96 ; 12.89)	5.11	°	(-0.43 ; 10.65)
6 14:00	-14.84	*	(-28.77 ; -0.92)	4.78	°	(-0.76 ; 10.32)
6 16:00	-5.19		(-19.11 ; 8.74)	2.83		(-2.71 ; 8.37)
6 18:00	-8.87		(-22.79 ; 5.06)	1.87		(-3.68 ; 7.42)
6 20:00	-11.03		(-24.95 ; 2.89)	3.53	°	(-0.39 ; 7.45)
7 10:00	4.32		(-9.61 ; 18.24)	0.11		(-5.43 ; 5.65)
7 12:00	-0.84		(-14.76 ; 13.08)	0.50		(-5.04 ; 6.05)
7 14:00	-9.56		(-23.48 ; 4.36)	2.50		(-3.04 ; 8.05)
7 16:00	-0.61		(-14.53 ; 13.31)	1.16		(-4.38 ; 6.70)
7 18:00	-6.06		(-19.98 ; 7.86)	-0.42		(-5.96 ; 5.13)
7 20:00	-1.10		(-15.02 ; 12.83)	1.65		(-2.26 ; 5.56)
**Day # Experiment**						
5 2	12.34	°	(-1.59 ; 26.26)	5.14	°	(-0.41 ; 10.70)
5 3	12.86	°	(-1.14 ; 26.86)	6.10	*	(-0.12 ; 12.32)
6 2	12.18	°	(-1.75 ; 26.10)	2.49		(-1.44 ; 6.42)
6 3	-2.45		(-16.44 ; 11.55)	8.04	*	(2.48 ; 13.61)
7 2	-8.10		(-22.03 ; 5.82)	-0.81		(-4.74 ; 3.12)
7 3	-8.18		(-22.18 ; 5.81)	4.79	°	(-0.77 ; 10.36)
**Time # Experiment**						
10:00 2	20.50	*	(6.58 ; 34.42)	1.48		(-4.06 ; 7.03)
10:00 3	20.78	*	(6.78 ; 34.78)	2.84		(-3.38 ; 9.07)
12:00 2	23.57	*	(9.65 ; 37.49)	0.10		(-5.45 ; 5.65)
12:00 3	29.54	*	(15.54 ; 43.54)	0.92		(-5.32 ; 7.16)
14:00 2	-2.31		(-16.23 ; 11.62)	4.77	°	(-0.78 ; 10.32)
14:00 3	16.23	*	(2.24 ; 30.23)	2.95		(-3.29 ; 9.19)
16:00 2	39.75	*	(25.83 ; 53.67)	4.51		(-1.04 ; 10.06)
16:00 3	22.36	*	(8.36 ; 36.36)	3.71		(-2.51 ; 9.93)
18:00 2	9.21		(-4.71 ; 23.14)	2.46		(-3.10 ; 8.01)
18:00 3	17.85	*	(3.85 ; 31.85)	3.63		(-2.63 ; 9.88)
20:00 2	7.74		(-6.18 ; 21.66)	-0.08		(-4.01 ; 3.84)
20:00 3	(n.e.)			(n.e.)		
**Day # Time # Experiment**						
5 10:00 2	-45.05	*	(-64.74 ; -25.36)	-4.92		(-11.71 ; 1.88)
5 10:00 3	-43.88	*	(-63.67 ; -24.09)	-10.10	*	(-17.47 ; -2.73)
5 12:00 2	-11.01		(-30.70 ; 8.68)	-3.62		(-10.41 ; 3.17)
5 12:00 3	-8.85		(-28.64 ; 10.95)	-5.52		(-12.90 ; 1.86)
5 14:00 2	14.03		(-5.66 ; 33.72)	-5.82	°	(-12.62 ; 0.98)
5 14:00 3	2.22		(-17.57 ; 22.02)	-4.66		(-12.04 ; 2.72)
5 16:00 2	-47.41	*	(-67.10 ; -27.72)	-6.06	°	(-12.85 ; 0.74)
5 16:00 3	-21.75	*	(-41.55 ; -1.96)	-3.52		(-10.88 ; 3.84)
5 18:00 2	-15.29		(-34.98 ; 4.40)	-5.65		(-12.45 ; 1.14)
5 18:00 3	-16.01		(-35.81 ; 3.78)	-3.41		(-10.81 ; 3.99)
5 20:00 2	-20.82	*	(-40.51 ; -1.13)	-6.75	*	(-12.30 ; -1.21)
5 20:00 3	(n.e.)			(n.e.)		
6 10:00 2	-18.54	°	(-38.23 ; 1.15)	-3.30		(-8.84 ; 2.24)
6 10:00 3	-1.49		(-21.29 ; 18.30)	-6.82	*	(-13.64 ; 0.00)
6 12:00 2	-6.89		(-26.58 ; 12.80)	-1.36		(-6.90 ; 4.18)
6 12:00 3	3.97		(-15.83 ; 23.76)	-5.56		(-12.40 ; 1.27)
6 14:00 2	3.41		(-16.28 ; 23.10)	-4.16		(-9.71 ; 1.38)
6 14:00 3	16.13		(-3.66 ; 35.93)	-3.58		(-10.43 ; 3.27)
6 16:00 2	-35.14	*	(-54.83 ; -15.45)	-3.45		(-8.99 ; 2.09)
6 16:00 3	12.00		(-7.79 ; 31.80)	-4.06		(-10.89 ; 2.76)
6 18:00 2	-15.27		(-34.96 ; 4.42)	0.93		(-4.62 ; 6.48)
6 18:00 3	21.53	*	(1.74 ; 41.33)	-5.02		(-11.86 ; 1.83)
6 20:00 2	1.74		(-17.95 ; 21.43)	(n.e.)		
6 20:00 3	(n.e.)			(n.e.)		
7 10:00 2	1.42		(-18.27 ; 21.11)	0.92		(-4.62 ; 6.46)
7 10:00 3	-0.08		(-19.88 ; 19.71)	-4.21		(-11.04 ; 2.61)
7 12:00 2	9.73		(-9.96 ; 29.42)	1.58		(-3.97 ; 7.12)
7 12:00 3	1.12		(-18.68 ; 20.91)	-3.55		(-10.40 ; 3.29)
7 14:00 2	25.89	*	(6.20 ; 45.58)	-2.01		(-7.55 ; 3.53)
7 14:00 3	9.93		(-9.86 ; 29.73)	-1.76		(-8.60 ; 5.09)
7 16:00 2	-12.49		(-32.20 ; 7.23)	-3.55		(-9.09 ; 1.99)
7 16:00 3	6.57		(-13.23 ; 26.36)	-3.33		(-10.15 ; 3.49)
7 18:00 2	14.91		(-4.81 ; 34.62)	-0.40		(-5.95 ; 5.14)
7 18:00 3	11.09		(-8.70 ; 30.89)	-2.16		(-9.01 ; 4.69)
7 20:00 2	4.91		(-14.78 ; 24.60)	(n.e.)		
7 20:00 3	(n.e.)			(n.e.)		
**Constant**	133.35	*	(125.81 ; 140.90)	15.49	*	(10.23 ; 20.74)
**Sigma_u**	**10.28**			**2.79**		
**Sigma_e**	**24.60**			**6.89**		
**ρ**	**0.1487**			**0.1408**		
**R^2_{Within}**	**18.84%**			**8.17%**		
**$R^2_{Between}$**	**6.15%**			**8.95%**		
**R^2**	**16.80%**			**8.40%**		

† light-off VMR restricted to the interval [-25;50]; * $P \leq 0.05$; ° $0.05 < P \leq 0.10$; a#b means interaction between a and b variables; (Ref.) means reference category; ρ is the fraction of the error variance due to the differences between individuals (Intraclass Correlation of the error); R^2_{Within}, $R^2_{Between}$ and R^2 and are the within, between and global correlations squared expressed in percentages; n.e. means not estimable.

Table S2. Pairwise comparisons of predictive margins of BLA for age. Differences and P-values corrected using the Bonferroni method to account for multiple comparisons.

**Age**			**A**		
			6	7	8
		**Predictive Margins**	**141.16**	**131.06**	**127.91**
**B**	5	**132.39**	**8.77**† 0.000*	**-1.33** 1.000	**-4.48** 0.000*
	6	**141.16**		**-10.10** 0.000*	**-13.25** 0.000*
	7	**131.06**			**-3.15** 0.031*

Note. † Difference between the predictive margins (A-B); * P ≤ 0.05; ° 0.05 < P ≤ 0.10; Shaded cells indicate non-statistically significant differences.

Table S3. Pairwise comparisons of predictive margins of BLA for time of the day. Differences and P-values corrected using the Bonferroni method to account for multiple comparisons.

Time of the day			A					
			10:00	12:00	14:00	16:00	18:00	20:00
		**Predictive Margins**	**137.06**	**139.87**	**135.18**	**140.18**	**124.42**	**115.58**
B	8:00	**133.81**	**3.25**† 0.536	**6.06** 0.001*	**1.37** 1.000	**6.37** 0.000*	**-9.39** 0.000*	**-18.23** 0.000*
	10:00	**137.06**		**2.81** 1.000	**-1.88** 1.000	**3.12** 0.672	**-12.64** 0.000*	**-21.48** 0.000*
	12:00	**139.87**			**-4.69** 0.027*	**0.31** 1.000	**-15.45** 0.000*	**-24.29** 0.000*
	14:00	**135.18**				**5.00** 0.012*	**-10.76** 0.000*	**-19.60** 0.000*
	16:00	**140.18**					**-15.76** 0.000*	**-24.60** 0.000*
	18:00	**124.42**						**-8.84** 0.000*

Note. † Difference between the predictive margins (A-B); * P ≤ 0.05; ° 0.05 < P ≤ 0.10; Shaded cells indicate non-statistically significant differences.

Table S4. Pairwise comparisons of predictive margins of BLA for time of the day and age. Differences and P-values corrected using the Bonferroni method to account for multiple comparisons.

DAY 5		Time of the day		A					
				10:00	12:00	14:00	16:00	18:00	20:00
			Predictive Margins	144.17	135.89	134.83	136.02	119.53	115.10
	B	8:00	135.47	**8.70**†<br>0.058°	**0.42**<br>1.000	**-0.64**<br>1.000	**0.55**<br>1.000	**-15.94**<br>0.000*	**-20.37**<br>0.000*
	B	10:00	144.17		**-8.28**<br>0.094°	**-9.34**<br>0.028*	**-8.15**<br>0.107	**-24.64**<br>0.000*	**-29.07**<br>0.000*
	B	12:00	135.89			**-1.06**<br>1.000	**0.13**<br>1.000	**-16.36**<br>0.000*	**-20.79**<br>0.000*
	B	14:00	134.83				**1.19**<br>1.000	**-15.30**<br>0.000*	**-19.73**<br>0.000*
	B	16:00	136.02					**-16.49**<br>0.000*	**-20.92**<br>0.000*
	B	18:00	119.53						**-4.43**<br>1.000
DAY 6			Predictive Margins	142.23	151.28	141.29	146.15	132.97	122.32
	B	8:00	145.64	**-3.41**†<br>1.000	**5.64**<br>1.000	**-4.35**<br>1.000	**0.51**<br>1.000	**-12.67**<br>0.000*	**-23.32**<br>0.000*
	B	10:00	142.23		**9.05**<br>0.039*	**-0.94**<br>1.000	**3.92**<br>1.000	**-9.26**<br>0.031*	**-19.91**<br>0.000*
	B	12:00	151.28			**-9.99**<br>0.012*	**-5.13**<br>1.000	**-18.31**<br>0.000*	**-28.96**<br>0.000*
	B	14:00	141.29				**4.86**<br>1.000	**-8.32**<br>0.090°	**-18.97**<br>0.000*
	B	16:00	146.15					**-13.18**<br>0.000*	**-23.83**<br>0.000*
	B	18:00	132.97						**-10.65**<br>0.026*
DAY 7			Predictive Margins	134.00	138.25	134.20	138.63	124.57	114.51
	B	8:00	127.76	**6.24**<br>0.673	**10.49**<br>0.007*	**6.44**<br>0.566	**10.87**<br>0.004*	**-3.19**<br>1.000	**-13.25**<br>0.001*
	B	10:00	134.00		**4.25**<br>1.000	**0.20**<br>1.000	**4.63**<br>1.000	**-9.43**<br>0.025*	**-19.49**<br>0.000*
	B	12:00	138.25			**-4.05**<br>1.000	**0.38**<br>1.000	**-13.68**<br>0.000*	**-23.74**<br>0.000*
	B	14:00	134.20				**4.43**<br>1.000	**-9.63**<br>0.020*	**-19.69**<br>0.000*
	B	16:00	138.63					**-14.06**<br>0.000*	**-24.12**<br>0.000*
	B	18:00	124.57						**-10.06**<br>0.050*
DAY 8			Predictive Margins	127.84	134.06	130.42	139.92	120.62	110.40
	B	8:00	126.37	**1.47**<br>1.000	**7.69**<br>0.174	**4.05**<br>1.000	**13.55**<br>0.000*	**-5.75**<br>1.000	**-15.97**<br>0.000*
	B	10:00	127.84		**6.22**<br>0.686	**2.58**<br>1.000	**12.08**<br>0.001*	**-7.22**<br>0.274	**-17.44**<br>0.000*
	B	12:00	134.06			**-3.64**<br>1.000	**5.86**<br>0.922	**-13.44**<br>0.000*	**-23.66**<br>0.000*
	B	14:00	130.42				**9.50**<br>0.023*	**-9.80**<br>0.016*	**-20.02**<br>0.000*
	B	16:00	139.92					**-19.30**<br>0.000*	**-29.52**<br>0.000*
	B	18:00	120.62						**-10.22**<br>0.042*

Note. † Difference between the predictive margins (A-B); * P ≤ 0.05; ° 0.05 < P ≤ 0.10; Shaded cells indicate non-statistically significant differences.

Table S5. Pairwise comparisons of predictive margins of light-off VMR for age. Differences and P-values corrected using the Bonferroni method to account for multiple comparisons.

Age			A		
			6	7	8
		**Predictive Margins**	**13.42**	**13.07**	**12.13**
**B**	5	**12.95**	**0.47**[†]<br>0.839	**0.12**<br>1.000	**-0.82**<br>0.066°
	6	**13.42**		**-0.35**<br>1.000	**-1.29**<br>0.000*
	7	**13.07**			**-0.94**<br>0.024*

Note. [†] Difference between the predictive margins (A-B); * P ≤ 0.05; ° 0.05 < P ≤ 0.10; Shaded cells indicate non-statistically significant differences.

Table S6. Pairwise comparisons of predictive margins of light-off VMR for time of the day. Differences and P-values corrected using the Bonferroni method to account for multiple comparisons.

Time of the day			A					
			10:00	12:00	14:00	16:00	18:00	20:00
		**Predictive Margins**	**11.39**	**11.62**	**12.14**	**12.38**	**14.32**	**14.91**
**B**	8:00	**14.48**	**-3.09**† 0.000*	**-2.86** 0.000*	**-2.34** 0.000*	**-2.10** 0.000*	**-0.16** 1.000	**0.43** 1.000
	10:00	**11.39**		**0.23** 1.000	**0.75** 1.000	**0.99** 0.337	**2.93** 0.000*	**3.52** 0.000*
	12:00	**11.62**			**0.52** 1.000	**0.76** 1.000	**2.70** 0.000*	**3.29** 0.000*
	14:00	**12.14**				**0.24** 1.000	**2.18** 0.000*	**2.77** 0.000*
	16:00	**12.38**					**1.94** 0.000*	**2.53** 0.000*
	18:00	**14.32**						**0.59** 1.000

Note. † Difference between the predictive margins (A-B); * P ≤ 0.05; ° 0.05 < P ≤ 0.10; Shaded cells indicate non-statistically significant differences.

Table S7. Pairwise comparisons of predictive margins of light-off VMR for time of the day and age. Differences and P-values corrected using the Bonferroni method to account for multiple comparisons.

				A					
**DAY 5**	**Time of the day**			10:00	12:00	14:00	16:00	18:00	20:00
			**Predictive Margins**	**10.70**	**11.93**	**12.96**	**12.70**	**15.37**	**14.82**
	**B**	8:00	**13.45**	**-2.75†**<br>0.022*	**-1.52**<br>1.000	**-0.49**<br>1.000	**-0.75**<br>1.000	**1.92**<br>0.477	**1.37**<br>1.000
	**B**	10:00	**10.70**		**1.23**<br>1.000	**2.26**<br>0.122	**2.00**<br>0.305	**4.67**<br>0.000*	**4.12**<br>0.000*
	**B**	12:00	**11.93**			**1.03**<br>1.000	**0.77**<br>1.000	**3.44**<br>0.001*	**2.89**<br>0.038*
	**B**	14:00	**12.96**				**-0.26**<br>1.000	**2.41**<br>0.072°	**1.86**<br>0.950
	**B**	16:00	**12.70**					**2.67**<br>0.024*	**2.12**<br>0.466
	**B**	18:00	**15.37**						**-0.55**<br>1.000
**DAY 6**			**Predictive Margins**	**12.61**	**12.92**	**12.38**	**12.36**	**14.23**	**15.32**
	**B**	8:00	**15.30**	**-2.71†**<br>0.025*	**-2.38**<br>0.088°	**-2.92**<br>0.010*	**-2.94**<br>0.009*	**-1.07**<br>1.000	**0.02**<br>1.000
	**B**	10:00	**12.61**		**0.31**<br>1.000	**-0.23**<br>1.000	**-0.25**<br>1.000	**1.62**<br>0.971	**2.71**<br>0.074°
	**B**	12:00	**12.92**			**-0.54**<br>1.000	**-0.56**<br>1.000	**1.31**<br>1.000	**2.40**<br>0.206
	**B**	14:00	**12.38**				**-0.02**<br>1.000	**1.85**<br>0.508	**2.94**<br>0.034*
	**B**	16:00	**12.36**					**1.87**<br>0.473	**2.96**<br>0.032*
	**B**	18:00	**14.23**						**1.09**<br>1.000
**DAY 7**			**Predictive Margins**	**11.26**	**11.35**	**12.83**	**12.29**	**13.80**	**15.34**
	**B**	8:00	**n.e.**						
	**B**	10:00	**11.26**		**0.09**<br>1.000	**1.57**<br>1.000	**1.03**<br>1.000	**2.54**<br>0.041*	**4.08**<br>0.000*
	**B**	12:00	**11.35**			**1.48**<br>1.000	**0.94**<br>1.000	**2.45**<br>0.059°	**3.99**<br>0.000*
	**B**	14:00	**12.83**				**-0.54**<br>1.000	**0.97**<br>1.000	**2.51**<br>0.143
	**B**	16:00	**12.29**					**1.51**<br>1.000	**3.05**<br>0.021*
	**B**	18:00	**13.80**						**1.54**<br>1.000
**DAY 8**			**Predictive Margins**	**10.99**	**10.25**	**10.37**	**12.19**	**13.85**	**14.16**
	**B**	8:00	**n.e.**						
	**B**	10:00	**10.99**		**-0.74**<br>1.000	**-0.62**<br>1.000	**1.20**<br>1.000	**2.86**<br>0.011*	**3.17**<br>0.014*
	**B**	12:00	**10.25**			**0.12**<br>1.000	**1.94**<br>0.380	**3.60**<br>0.000*	**3.91**<br>0.001*
	**B**	14:00	**10.37**				**1.82**<br>0.570	**3.48**<br>0.001*	**3.79**<br>0.001*
	**B**	16:00	**12.19**					**1.66**<br>0.929	**1.97**<br>0.708
	**B**	18:00	**13.85**						**0.31**<br>1.000

Note. † Difference between the predictive margins (A-B); * P ≤ 0.05; ° 0.05 < P ≤ 0.10; n.e.: not estimable. Shaded cells indicate non-statistically significant differences.
